# Editorial: Long COVID-19: ultimate reasoning as a need for the search of proximate solutions

**DOI:** 10.3389/fcimb.2023.1227626

**Published:** 2023-06-05

**Authors:** Leo Pruimboom

**Affiliations:** ^1^Human Sciences, Pontifical University of Salamanca, Salamanca, Spain; ^2^Chair of Clinical Psychoneuroimmunology, University of Granada, Granada, Spain

**Keywords:** SARS-CoV-2, long COVID, lifestyle, evolutionary medicine, COVID prevention

Long COVID-19 has become the major burden of the still-active COVID-19 pandemic worldwide. Long COVID-19 and its symptoms are present in more than 45% of all COVID-19 survivors at 4 months post-infection. Symptoms vary between fatigue, muscle aches, depression, brain fog, and shortness of breath, in addition to many others. Mechanisms related to the development of long Covid-19 point to processes such as viral persistence, residual viral load, immunological disturbances including leptin and cytokine resistance, epigenetic mutations, tissue and organ damage, microvascular blood clothing, and iatrogenic factors (Pruimboom, 2020; Muskiet et al., 2022; Davis et al., 2023). This Research Topic focused on evolutionary reasoning as an explanation for the impact of a viral infection affecting any type of person and the proximal consequences of the infection in the long run. The topics of the manuscripts of this Research Topic range from quality of life to specific treatment options in people suffering from COVID-19 and the post-COVID-19 consequences.

Li et al. investigated the impact of the protective use of ursodeoxycholic acid (UDCA) against COVID-19 in patients with liver disease compared with a matched control group not receiving UDCA. The primary outcome was that UDCA therapy could possibly decrease COVID-19 infection risk, mitigate symptoms, and shorten the course in patients with chronic liver disease, with the latter having protective effects against long COVID-19. If further research shows that UDCA therapy could protect against COVID-19 severity and overall infection susceptibility, UDCA could even be used as preventive intervention for long COVID-19. A pre-print about the possible effect of UDCA on the recovery time of patients suffering from COVID-19 (Yu et al., 2023) supports the data of Li et al. Interestingly, the use of UDCA was already part of traditional Chinese medicine in the form of ‘yutan’, a dried powder preparation derived from the dried bile of adult bears and given to patients suffering from hepatobiliary disorders (Lazaridis et al., 2001). Not surprisingly, a cross-sectional study in 2023 (de-Lima et al., 2023) in 243 patients suffering from long COVID-19 showed altered liver functions mostly when they were hospitalized when suffering from acute COVID-19. Therefore, ancient evolutionary interventions offer proximate protection against long COVID-19 when suffering from an infection caused by SARS-COV-2.

The study of Bhattacharyya et al. highlighted the need for immediate increased attention toward the consequences of long COVID-19 worldwide. Their innovative study using statistics of sentiments of people expressed *via* Twitter accounts showed the influence of not knowing enough about long COVID-19 on emotions such as disgust, joy, sadness, surprise, trust, and negative and positive feelings. Negative emotions and depressive mood should be recognized as major risk factors for the development of long COVID-19, as shown by the study of Wang et al. (2022). Our generation has never experienced a pandemic before, and a lack of knowledge produces anxiety defined by ‘fear of the unknown’. Knowledge of the long-term effects of COVID-19 can possibly serve as a basis for the development of primary and secondary preventive interventions against viral infections and long-term consequences, as stated by Bhattacharyya et al. Emotional and psychosocial factors, which have been part of all human beings since the existence of homo sapiens, should be considered part of the etiology of long COVID-19 (Wang et al., 2022).

Liska et al. investigated the quality of life in patients suffering from long COVID-19 and compared this variable with healthy students. The outcome was that long-COVID-19 sufferers scored worse on all measured parameters of quality of life than the healthy control group. The authors claimed the need for the development of interventions that improve the quality of life in chronically affected individuals. Pain, fatigue, and mental health were all more present in the long-COVID group, and they were possibly related not only with viral infection but also with the possible anxiety and stress factors associated with isolation and fear of infection. Homo sapiens developed through multiple evolutionary pressure factors and their solutions, of which socializing is considered one of the most important behavioral traits of modern humans (Belsky et al., 1991; Lacruz et al., 2019).

He et al. hypothesized that SARS-COV-2 could possibly persist, together with other pathogens in different organs and tissues, through the forming of a biofilm. The authors proposed that long-COVID-19 symptoms of the lung could be explained by virus escape of the biofilm during immunocompromised periods. This could also explain the re-positivity and long-term positive nucleic acid testing during recovery from COVID-19. The capacity of SARS-COV-2 to form a biofilm with other pathogens is an ancient strategy of multiple bacteria and viruses to survive in a hostile host, and SARS-COV-2 is not different from other pathogens in exploiting survival strategies (Sentenac et al., 2022). Biofilm production can be influenced by several interventions, and therefore, if and when the hypothesis of He et al. is confirmed, preventive oral hygiene (Al-Bayaty et al., 2021), nose irrigation (Keen et al., 2010), and even therapeutic inhalable substances such as glutathione could be indicated for primary and secondary prevention of long COVID-19 (Ding et al., 2021). Biofilm formation is part of the evolution of most multi-cellular organisms, and beneficial biofilm formation in the human gut is essential to maintain not only gut but also systemic health (Penesyan et al., 2021; Sentenac et al., 2022; Li et al., 2023).

Wang et al. stated that long COVID should be considered a thrombotic sequela. Possible persistence of the virus causes chronic inflammation and continuous endothelial damage, both of which induce activity of the thrombotic mechanisms. Further complications of the increase of thrombotic activity in patients suffering from long COVID-19 include chronic hypoxia leading to capillary dysfunction and thrombosis. It may be obvious to consider thrombotic activity as a life-saving mechanism in situations such as wound healing and infection (Locatelli et al., 2021). Normally, thrombotic activity is time-restricted and part of a physiological wound healing response. SARS-COV-2 seems to have exploited another mechanism of the human immune system to its own benefit, producing long-lasting thrombotic activity that causes many of the symptoms related with long COVID-19. Here again, evolutionary reasoning is demonstrated as a way to find solutions for a new pandemic viral infection, using anti-thrombolytic interventions to decrease the long-term impact of COVID-19.

COVID-19 has had different effects on individuals living in developed countries compared with those living in developing countries such as Bangladesh. The study of Ahmed et al., based on self-reported data derived from a mixed qualitative and quantitative interview method, showed that a substantial number of individuals suffer long COVID after acute COVID-19 but lack proper support for their physical and mental wellbeing. The authors alluded to the need for quality studies in countries such as Bangladesh and the possibility of transferring knowledge and thus helping to fight the long-COVID pandemic in low- and middle-income countries (LMICs). Bangladesh, as one of the 74 poorest countries in the world, is only one example of those countries that are suffering the greatest consequences of a pandemic that has already lasted for more than 3 years. The study group of Ahmed et al. has the potential to emerge as a cohort with scope for future health and social interventions to design and deliver the required healthcare for “long COVID” in Bangladesh and by extension to other similar LMICs.

In summary, the results of the different studies and reviews included in our Research Topic make it clear that knowledge about the causes of and mechanisms behind long COVID-19, and the need for effective treatment options for people suffering from it, is increasing but absolutely not complete or satisfying. The quality of life of patients suffering from long COVID is severely affected, and the number of individuals suffering from it is still increasing. Not only is the individual health impact of great concern, but the global economic burden is another consequence that is no less important. We, the editors of this special issue, hope that the manuscripts included have added value to the research about long COVID-19 and that they will help to treat patients with long COVID successfully, but even more so to prevent its development in new patients.

## Author contributions

The author confirms being the sole contributor of this work and has approved it for publication.
